# Gut microbiota dysbiosis in people living with HIV who have cancer: novel insights and diagnostic potential

**DOI:** 10.3389/fimmu.2025.1598650

**Published:** 2025-08-25

**Authors:** Zhiman Xie, Qianqian Huang, Lemin Wen, Tingyan Luo, Sufang Ai, Guangjing Ruan, Ningmei Liu, Yiru Wei, Yingji Lan, Ping Cui, Qin Cao, Hao Liang, Li Ye, Jie Zhou, Jiegang Huang

**Affiliations:** ^1^ The Fourth People’s Hospital of Nanning & Nanning Infectious Diseases Hospital Affiliated to Guangxi Medical University, Nanning, Guangxi, China; ^2^ Guangxi Key Laboratory of AIDS Prevention and Treatment & School of Public Health, Guangxi Medical University, Nanning, Guangxi, China; ^3^ Guangxi Colleges and Universities Key Laboratory of Prevention and Control of Highly Prevalent Diseases, Guangxi Medical University, Nanning, Guangxi, China; ^4^ Life Science Institute, Guangxi Medical University, Nanning, Guangxi, China

**Keywords:** HIV/AIDS, pan-cancer, gut microbiota, machine learning, *Bacteroides*

## Abstract

**Background:**

People living with HIV(PLWH) are a high-risk population for cancer. We conducted a pioneering study on the gut microbiota of PLWH with various types of cancer, revealing key microbiota.

**Methods:**

We collected stool samples from 54 PLWH who have cancer (PLWH-C), including Kaposi’s sarcoma (KS, n=7), lymphoma (L, n=22), lung cancer (LC, n=12), and colorectal cancer (CRC, n=13), 55 PLWH who do not have cancer (PLWH-NC), and 49 people living without HIV (Ctrl). The gut microbiota in fecal samples was analyzed using 16S rRNA sequencing. We compared the microbial diversity among groups and identified key microbiota and Kyoto Encyclopedia of Genes and Genomes (KEGG) pathways using random forest. Furthermore, we analyzed the correlation between microbiota and KEGG pathways and constructed microbiota Receiver Operating Characteristic (ROC) diagnostic models.

**Results:**

Compared with PLWH-NC and Ctrl, PLWH with any type of cancer exhibited significantly lower alpha diversity and significant alterations in beta diversity of the gut microbiota. The significantly decreased abundance of *Bacteroides* and *Bacteroides vulgatus* in PLWH-C showed a negative correlation with the *Pathways in cancer pathway*, and a positive correlation with *Choline metabolism in cancer*, *Central carbon metabolism in cancer*, and *Proteoglycans in cancer pathways*. *Bacteroides* (AUC≥0.84) and *Bacteroides vulgatus* (AUC≥0.78) exhibited discriminatory diagnostic capabilities for PLWH-C in patients with different cancers compared with PLWH-NC and Ctrl.

**Discussion:**

We confirmed a more severe dysbiosis of the gut microbiota in PLWH with KS, L, LC, or CRC. *Bacteroides* may be associated with disruptions in cancer-related metabolic pathways and serve as diagnostic biomarkers for PLWH with various cancers.

## Introduction

The gut microbiota interacts with the host to participate in crucial metabolic processes within the human body. It provides various enzymes and metabolic pathways for specific metabolic processes, playing a vital role in maintaining gut homeostasis. An increasing body of research suggests that the gut microbiota plays a role in the pathogenesis of various diseases, including inflammatory bowel disease (1), diabetes (2), tumors (3), and HIV/AIDS (4). HIV/AIDS poses a significant threat to human health. Research indicates that during HIV infection, the gut mucosal barrier is disrupted. Early in infection, the virus rapidly attacks the gut-associated lymphoid tissue, which contains one of the highest concentrations of CD4^+^ T cells. These immune cells play a key role in maintaining gut barrier integrity and prevent bacterial translocation. As gut permeability increases, microbial translocation triggers chronic immune activation, which in turn accelerates HIV disease progression and causes a further reduction in CD4^+^ (5). Alpha diversity commonly measures the abundance and uniformity of microbial species within a single sample, whereas beta diversity measures the variation in microbial composition among samples. Both alpha diversity and beta diversity are reduced in the gut microbiota of People living with HIV(PLWH), who also show an increased abundance of *Prevotella* and a decreased abundance of *Bacteroides* (6).

With the widespread application of highly active antiretroviral therapy (HAART), AIDS has evolved into a chronic disease. HAART has extended the survival of PLWH; however, due to incomplete immune recovery, there is an increased incidence of cancer. Moreover, individuals with both HIV and cancer experience more severe clinical symptoms and lower survival rates (7, 8). HIV infection combined with tumors has become one of the leading causes of death among PLWH. The 19th European Congress on AIDS indicated that the incidences of both AIDS-defining cancers and non-AIDS-defining cancers (NADCs) declined from 1997 to 2018, but they remain higher than in the general population. Furthermore, the number of cancer cases among male individuals living with HIV was nearly double that of uninfected males. Age-related cancers have increased among infected individuals (9). AIDS-defining cancers include Kaposi’s sarcoma (KS), non-Hodgkin lymphoma, and invasive cervical cancer. NADCs include Hodgkin lymphoma, lung cancer (LC), and colorectal cancer (CRC), etc. (10). The gut microbiota is a risk or preventive factor for various diseases. An increase in the abundance of *Fusobacterium* and *Sphingomonas* has been observed in CRC (11); specific gut microbiota features for predicting early-stage lung cancer were established based on 13 high-precision OTU biomarkers (12); and in PLWH with oral KS, a decrease in the abundance of the genera *Aggregibacter* and *Lautropia* was observed (13).

The gut microbiota is closely associated with both HIV and cancer, potentially serving as a novel target for therapeutic interventions. However, the characteristics of the gut microbiota and key biomarkers of PLWH with various cancers have not been investigated. This study analyzed the characteristics of the gut microbiota of PLWH who have cancer (PLWH-C), including KS, lymphoma (L), LC, and CRC, and identified specific microbial community characteristics that may be helpful in the diagnosis of PLWH-C. This exploration provides guidance for the investigation of potential biomarkers for the diagnosis, treatment, and prognosis of HIV with concomitant cancer.

## Methods

### Subject recruitment and sample collection

We conducted a cross-sectional study. PLWH were recruited from the Fourth People’s Hospital of Nanning (Nanning, Guangxi, China), and people living without HIV (Ctrl) were enrolled from the health examination department of the First Affiliated Hospital of Guangxi Medical University. In simple terms, individuals meeting the following criteria were included in this study: PLWH were required to meet the diagnostic criteria outlined in the Chinese guidelines for the diagnosis and treatment of HIV/AIDS (2021) (14); PLWH who have cancer were those diagnosed with KS, L, LC, or CRC through pathological examination; PLWH who do not have cancer (PLWH-NC) and Ctrl were identified through clinical physician screening, with no previous history of cancer and currently presenting no symptoms or signs related to tumor diseases. The exclusion criteria for the subjects were as follows: history of antibiotic or other medication use within the past 2 weeks that affects the gut microbiota; PLWH-NC and Ctrl had factors that may affect the gut microbiota, such as hypertension, diabetes mellitus, coronary artery disease, chronic kidney disease, and pregnancy. This study was approved by the Ethics Committee of the Fourth People’s Hospital of Nanning (Approval number: [2022]45). All participants signed an informed consent form before participating in the study.

We collected demographic and clinical information, as well as stool samples, from the subjects. Stool samples were collected using tubes containing stool DNA stabilizers (STRATEC stool collection tubes with stool DNA stabilizer, Germany), which can lyse and release DNA from stool microbiota and maintain DNA stability at room temperature for up to 3 months. Generally, stool samples were transported to the laboratory on ice within 6 hours of collection and then stored at -80°C. Every 3 months, a batch of stool samples was transported on dry ice to the company (Majorbio BioPharm Technology Co., Ltd., Shanghai, China) for sequencing to avoid technical and operational variability caused by multiple sequencing runs.

### 16S rRNA sequencing

DNA was extracted from stool samples using an EZNA^®^ DNA Kit (Omega Bio-tek, Norcross, GA, USA). Primers 338F (5’-ACTCCTACGGGAGGCAGCAG-3’) and 806R (5’-GGACTACHVGGGTWTCTAAT-3’) were used to amplify the V3-V4 variable region of the 16S rRNA gene by PCR. The amplified PCR products were then purified, recovered, and quantified. The NEXTFLEX Rapid DNA-Seq Kit was used to construct the library. Sequencing was performed on an Illumina MiSeq PE300 platform (Majorbio BioPharm Technology Co., Ltd., Shanghai, China).

### 16S rRNA data processing and differential bacteria identification

Fastp 15 was used to control the quality of the original sequencing sequence. Microbiota bioinformatics was performed using QIIME 2 (version 2021.11) (15). Quality filtering of raw sequences using the q2-demux plugin, followed by DADA2 17 (via q2-dada2) for denoising and generation of amplicon sequence variants (ASV). All ASVs were aligned with Mafft (q2-alignment) and used to construct a phylogenetic tree with FastTree2 (q2-phylogeny). The species annotation of ASV was performed using silva-138 99% OTU reference sequences pretrained with the classify-sklearn Naive Bayes classifier and the q2-feature-classifier plugin. Based on the microbial sequence and ASV table, we used PICRUSt2 (16) to predict KEGG functional pathways and generate a table of relative functional abundances.

The generated ASV table, phylogenetic tree, and species annotation files were imported into R 4.2.2, and the microeco package was used to normalize the features to enable comparison across samples with the same sequencing depth. The microeco package was used to calculate microbial alpha and beta diversity. The alpha diversity indices included community richness indices (Observed, Chao1, and ACE), community diversity and evenness indices (Fisher, Shannon, Simpson, and Invsimpson), and the phylogenetic diversity index (PD). Principal Coordinate Analysis (PCoA) based on the Bray–Curtis distance algorithm was conducted to assess the similarity of beta diversity among samples. We used the feature screening tool Random Forest analysis in the microeco package to identify differential bacteria and Kyoto Encyclopedia of Genes and Genomes (KEGG) functional pathways among groups. The Venn diagram was used to identify overlapping factors between groups. Cytoscape 3.7.1 software was used to draw the correlation network diagram between bacterial abundance and clinical indicators. GraphPad Prism (version 6.01) was used to construct Receiver Operating Characteristic (ROC) diagnostic models.

### Statistical analysis

Statistical analysis was performed using R 4.2.2. Variables with missing values of less than 20% were imputed using multiple imputation. For data that met normality were described using the mean ± standard deviation and analyzed them using the Student’s t-test. For data that did not meet the normality criteria were described using median (interquartile range) and analyzed them using the Mann–Whitney U test. The count data were described using the number of cases and analyzed them using the chi-square test. The nonparametric PERMANOVA test was used to assess significant differences in gut microbiota structure between groups. For data that met normality, the Pearson correlation coefficients between indicators were calculated. For data that did not meet normality, we calculated the Spearman correlation coefficients between indicators. All tests were two-sided, and *P*<0.05 was considered statistically significant.

## Results

### Characteristics of the subjects

A total of 54 subjects were included in the PLWH-C group, consisting of PLWH-KS (n=7), PLWH-L (n=22), PLWH-LC (n=12), and PLWH-CRC (n=13). The PLWH-NC and Ctrl groups included 55 and 49 subjects, respectively. There were no statistically significant differences in antiretroviral therapy (ART) regimens between PLWH-C and PLWH-NC. There were no significant differences in the use of antibiotics or other drugs that may affect the gut microbiota within the past two weeks (*P*>0.05). The baseline characteristics of the subjects were shown in **Table 1**. Additionally, most PLWH receiving ART had viral loads below 20 copies/mL, while newly diagnosed patients in the PLWH-NC group mostly had viral loads above 20 copies/mL (**Supplementary Table 1**). However, due to approximately 35% missing data, further analysis could not be performed.

**Table 1:** Characteristics of subjects.

Characteristics	PLWH-C^a^(n=54)					PLWH-NC^b^ n=55	Ctrl^c^ n=49	*P*-value
	KS^d^, n=7	L^e^, n=22	LC^f^, n=12	CRC^g^, n=13	All			
Gender								0.728
Male	6	18	9	9	42	45	37	
Female	1	4	3	4	12	10	12	
Age	40.00 ± 15.29	53.14 ± 11.54	64.08 ± 7.90	60.92 ± 10.39	55.74 ± 13.21	42.07 ± 12.37	42.00 (33.00, 54.50)	<0.001
BMI	20.15 ± 2.83	19.62 (16.94, 22.66)	23.44(20.52, 26.14)	19.56 ± 1.88	20.89 ± 3.52	20.75 (19.03, 22.10)	23.44 ± 2.85	<0.001
MSM								0.011
Yes	0	0	0	0	0	5	0	
No	7	22	12	13	54	50	49	
CD4^+^	48.00(36.00, 120.00)	132.00(95.25, 227.25)	407.00(307.61, 641.25)	284.38 ± 183.58	195.00(102.75, 349.25)	410.40 ± 219.64	–	<0.001
ART regimen								0.129
Non-ART	4	16	10	8	38	31	–	
NRTI-containing regimen	3	6	2	5	16	24	–	
NRTI-sparing regimen	0	0	0	0	0	0	–	
Antibiotics or other drugs that affect the microbiota								0.660
Yes	0	0	0	1	1	0	0	
No	7	22	12	12	53	55	49	

^a^PLWH-C, People living with HIV who have cancer.

^b^PLWH-NC, People living with HIV who do not have cancer.

^c^Ctrl, People living without HIV.

^d^KS, Kaposi's sarcoma.

^e^L,Lymphoma.

^f^LC, Lung cancer.

^g^RC, Colorectal cancer.

### PLWH with cancer exhibited a more significant dysbiosis of the gut microbiota

**Figure 1:**
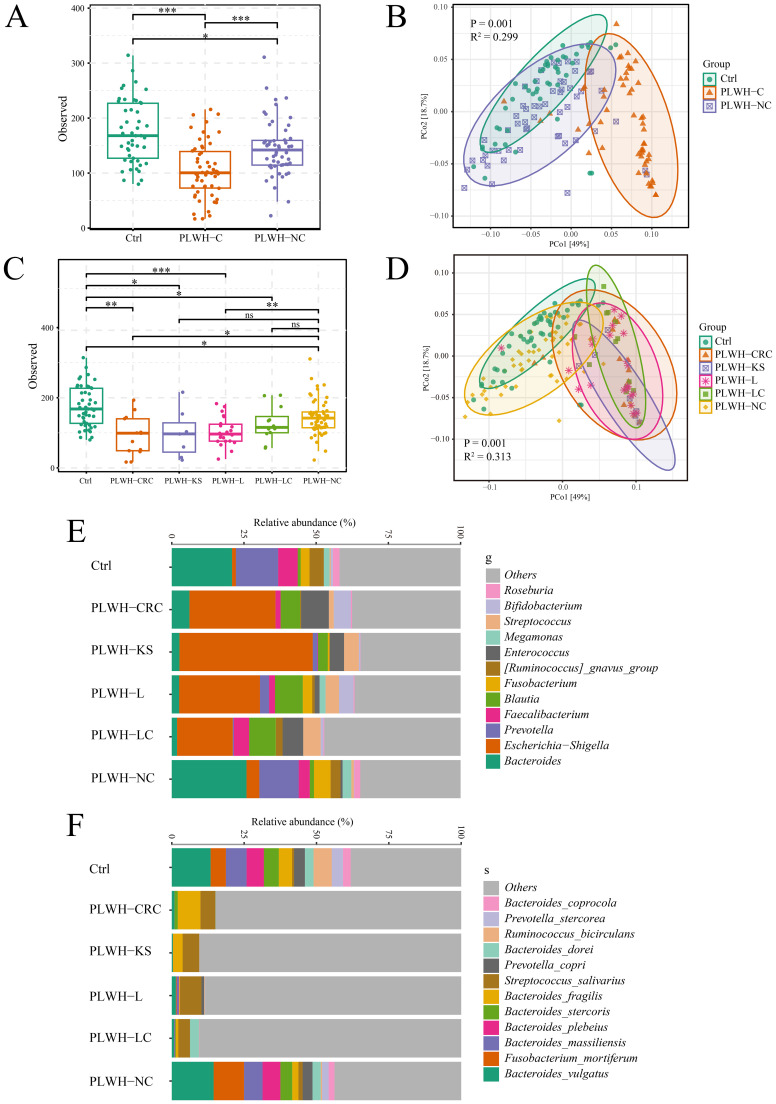
PLWH-C exhibited profound dysbiosis of gut microbiota. Comparison chart of alpha **(A)** and beta **(B)** diversity between among PLWH-C, PLWH-NC, and Ctrl. Comparison chart of alpha **(C)** and beta **(D)** diversity between among PLWH-KS, PLWH-L, PLWH-LC, PLWH-CRC, PLWH-NC, and Ctrl. Stacking chart of percentage at the level of genera **(E)** and species **(F)** of bacteria. *P<0.05, **P<0.01, ***P<0.001. ns, not statistically significant.

We initially compared the diversity of the gut microbiota among PLWH-C, PLWH-NC, and Ctrl groups. The results showed a decrease in gut microbiota alpha diversity in both PLWH-C and PLWH-NC compared with Ctrl (**Figure 1A**), with a distinct distribution of PLWH in the PCoA plots compared with Ctrl (**Figure 1B**). These alterations were more pronounced in PLWH-C, where alpha diversity was significantly lower than in PLWH-NC and Ctrl (**Figure 1A**). The samples showed distinct separation from both PLWH-NC and Ctrl in the PCoA plots (**Figure 1B**). Therefore, we observed distinct characteristics among the different cancer types. The results indicated that the alpha diversity of PLWH-KS, PLWH-L, PLWH-LC, and PLWH-CRC was lower than that of PLWH-NC and Ctrl (**Figure 1C**). The samples were also significantly separated from PLWH-NC and Ctrl in PCoA coordinates (**Figure 1D**). These findings indicate that HIV infection alters the diversity of the gut microbiota in the human body, and KS, L, LC, and CRC further intensify these changes in gut microbiota diversity.

Furthermore, the composition of the gut microbiota in the study subjects was illustrated using a percentage stacked bar chart. At the genus level, it was evident that compared with PLWH-NC and Ctrl, the proportions of *Bacteroides*, *Prevotella*, and others were reduced, whereas the proportions of *Escherichia-Shigella*, *Blautia*, and others were increased in PLWH-KS, PLWH-L, PLWH-LC, and PLWH-CRC patients (**Figure 1E**). Random forest analysis confirmed significant differences among the six groups of 178 genera, including *Bacteroides*, *Prevotella*, *Escherichia-Shigella*, and *Blautia* (**Supplementary Table 2**). At the species level, compared to PLWH-NC and Ctrl, the proportions of *Bacteroides vulgatus*, *Bacteroides stercoris*, and *Bacteroides plebeius* were decreased in PLWH-KS, PLWH-L, PLWH-LC, and PLWH-CRC patients. In contrast, the proportion of *Streptococcus salivarius* increased (**Figure 1F**). Random forest analysis also confirmed significant differences among the six groups for 114 species, including *Bacteroides vulgatus*, *Bacteroides stercoris*, *Bacteroides plebeius*, and *Streptococcus salivarius* (**Supplementary Table 3**).

### Dysbiosis of the gut microbiota is associated with cancer metabolic pathways in PLWH who have cancer

Indeed, closely related microorganisms generally exhibit similar genetic compositions and metabolic functions. We employed the PICRUSt2 (16) algorithm to compare the 16S rRNA gene sequences of unknown microorganisms with those of known genomic databases, thereby inferring their potential functional characteristics. Subsequently, differential pathways among groups were identified using Random Forest analysis, a widely used method for the differential analysis of microbiome data. The proposed method integrates ensemble learning by constructing a large number of decision trees. Each tree uses randomly selected features of the microbial community to make grouping judgments, and the results of all trees are ultimately combined to assess the contribution of each genus to classification decisions (17). The contribution of each genus to the sample classification was quantified using the Gini index or feature importance rankings, thereby identifying the most relevant differential taxa associated with the experimental grouping. A total of 360 differential KEGG functional pathways were identified among Ctrl, PLWH-NC, PLWH-KS, PLWH-L, PLWH-LC, and PLWH-CRC (**Supplementary Table 4**). Among these pathways, we identified significant intergroup differences in several cancer-related pathways, including *Pathways in cancer* and metabolism-related pathways, such as central *carbon metabolism in cancer*, *Choline metabolism in cancer*, and *Proteoglycans in cancer*. Furthermore, we analyzed the correlations between the abundances of 178 differential genera and 114 differential species with those of the four aforementioned pathways.

**Figure 2:**
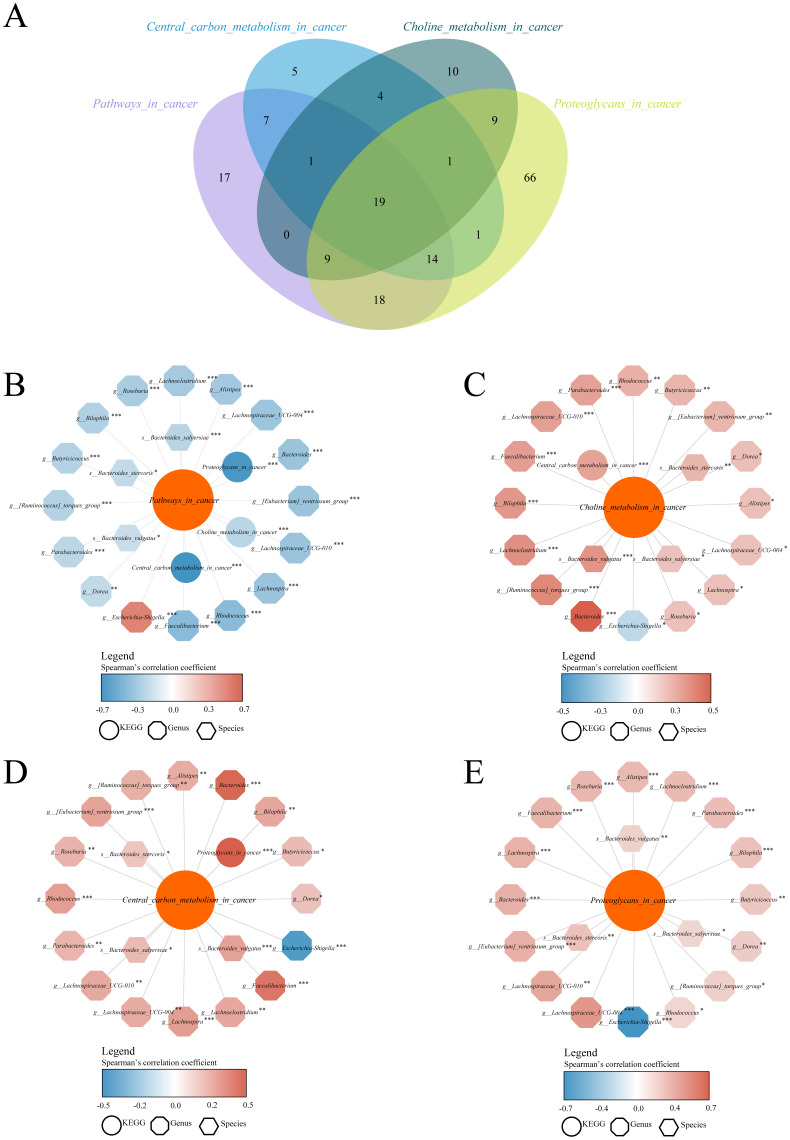
Dysbiosis of the gut microbiota in PLWH-C was associated with cancer metabolic pathways. Identify intersecting bacteria significantly associated with multiple cancer pathways using the Venn diagram **(A)**. Correlation analysis between intersecting bacteria and the KEGG pathways of *Pathways in cancer* **(B)**, *Choline metabolism in cancer* **(C)**, *Central carbon metabolism in cancer* **(D)**, and *Proteoglycans in cancer* **(E)** *pathways.* *P<0.05, **P<0.01, ***P<0.001.

Using Spearman correlation analysis, we identified 85 genera or species with significant correlations with the *pathways in the cancer pathway* (**Supplementary Table 5**), 52 genera or species with significant correlations with the *Central carbon metabolism pathway* (**Supplementary Table 6**), 53 genera or species with significant correlations with the *choline metabolism pathway* (**Supplementary Table 7**), and 137 genera or species with significant correlations with the *proteoglycans pathway* (**Supplementary Table 8**). Bacteria with significant correlations with all of the multiple cancer pathways were identified through Venn diagram analysis, including 19 intersecting genera, such as *Bacteroides*, *Escherichia-Shigella, and Bacteroides stercoris*, which were significantly correlated with the abundance of all four cancer metabolic pathways in Spearman correlation analyses at *P*<0.05 (**Figure 2A**, **Supplementary Table 9**). Through the correlation network diagram, we observed that among these 19 intersecting genera, except for *Escherichia-Shigella*, all were significantly negatively correlated with *Pathways in cancer* (**Figure 2B**) and significantly positively correlated with *Choline metabolism in cancer* (**Figure 2C**), *Central carbon metabolism in cancer* (**Figure 2D**), and *Proteoglycans in cancer* (**Figure 2E**).

### *Bacteroides* demonstrate promise as a diagnostic biomarker of HIV-associated cancers

**Figure 3:**
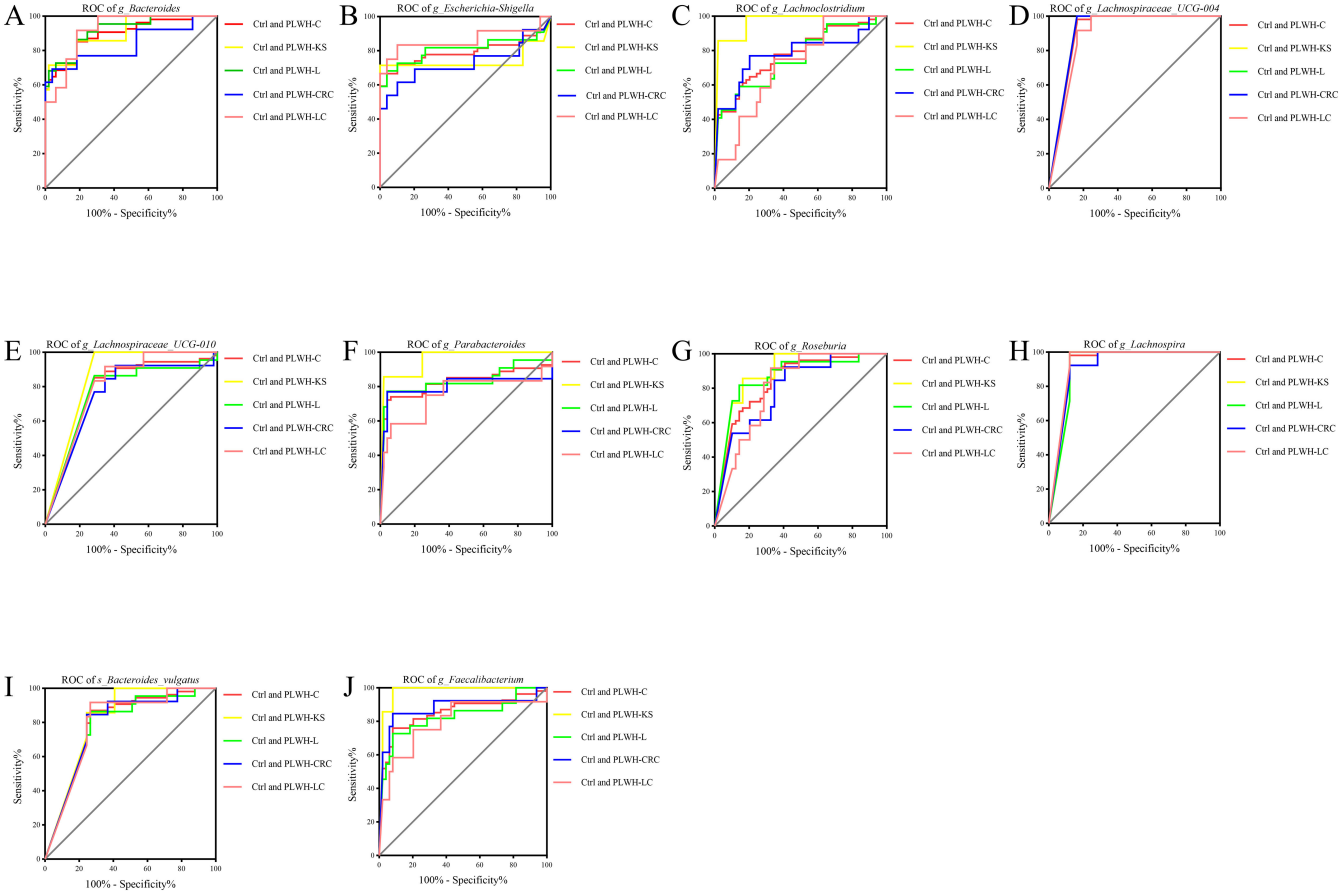
Gut microbiota associated with cancer pathways be used to distinguish PLWH-C from Ctrl. Ten genera or species, including *Bacteroides* **(A)**, *Escherichia-Shigella* **(B)**, *Lachnoclostridium* **(C)**, *Lachnospiraceae_UCG-004* **(D)**, *Lachnospiraceae_UCG-010* **(E)**, *Parabacteroides* **(F)**, *Roseburia* **(G)**, *Lachnospira* **(H)**, *Bacteroides vulgatus* **(I)** and *Faecalibacterium* **(J)**, could be used for diagnosing PLWH-C and Ctrl, as well as PLWH-KS and Ctrl, PLWH-L and Ctrl, PLWH-CRC and Ctrl, PLWH-LC and Ctrl (All *P*<0.05, AUC>0.70).

**Figure 4:**
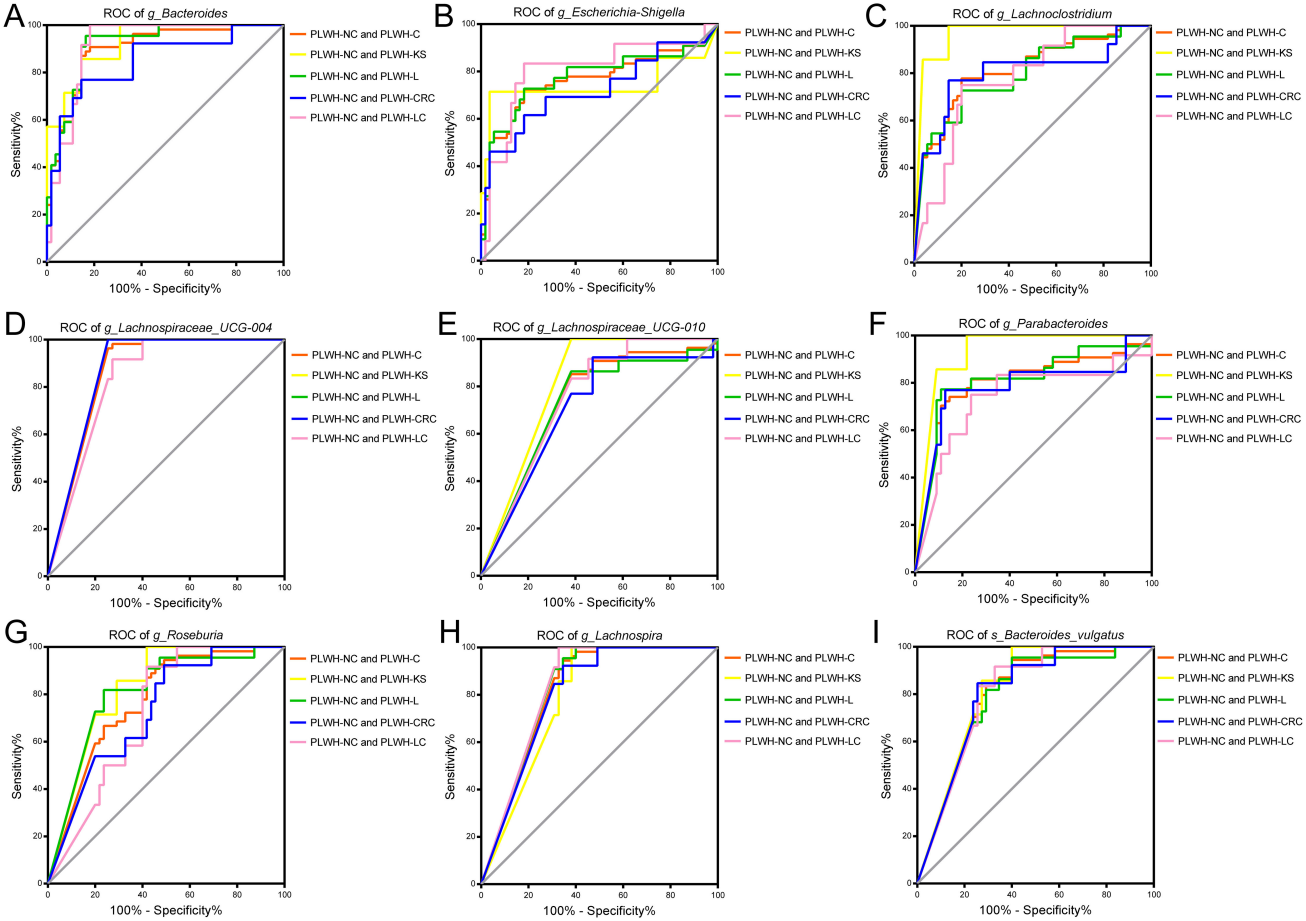
Gut microbiota associated with cancer pathways be used to distinguish PLWH-C from PLWH-NC. Nine genera or species, including *Bacteroides* **(A)**, *Escherichia-Shigella* **(B)**, *Lachnoclostridium* **(C)**, *Lachnospiraceae_UCG-004* **(D)**, *Lachnospiraceae_UCG-010* **(E)**, *Parabacteroides* **(F)**, *Roseburia* **(G)**, *Lachnospira* **(H)**, and *Bacteroides vulgatus* **(I)**, could be used for diagnosing PLWH-C and PLWH-NC, as well as PLWH-KS and PLWH-NC, PLWH-L and PLWH-NC, PLWH-CRC and PLWH-NC, PLWH-LC and PLWH-NC (All *P*<0.05, AUC>0.70).

ROC models were constructed using the abundance profiles of the 19 intersecting genera identified to diagnose people without HIV, PLWH-C, and PLWH-NC. The results showed that 9 genera or species, including *Bacteroides, Escherichia-Shigella, Lachnospira, Lachnospiraceae UCG 004, Lachnospiraceae UCG 010, Lachnoclostridium, Parabacteroides*, *Roseburia*, and *Bacteroides vulgatus*, could serve as potential diagnostic markers for PLWH-C (including PLWH-KS, PLWH-L, PLWH-LC, and PLWH-CRC) when compared with PLWH-NC and Ctrl individuals (**Figures 3A–I**, **4A–I**). There is also a genus, *Faecalibacterium*, that can only be used to diagnose PLWH-C and Ctrl (All *P*<0.05, AUC>0.70; **Figure 3**, **Supplementary Table 11**). Among them, *Bacteroides* exhibited the best diagnostic performance for all the diagnostic combinations (AUC≥0.84; **Figures 3A**, **4A**, **Supplementary Tables 10**, **11**). Additionally, the subspecies *Bacteroides vulgatus* was the only key species that could be used for all the diagnostic combinations (AUC≥0.78; **Figures 3I**, **4I**, **Supplementary Tables 9**, **10**).

## Discussion

The incidence of both AIDS-defining cancers and NADCs has decreased, but approximately 10%-20% of PLWH still succumb to cancer (7). Dysbiosis of the gut microbiota has been found to be associated with HIV (4, 6) or cancer (3, 12, 18), but few studies have explored the diversity of the gut microbiota in PLWH who have cancer. In this study, we explored the gut microbiota characteristics of PLWH-KS, PLWH-CRC, PLWH-LC, and PLWH-L for the first time in a microbial spectrum associated with HIV-related cancers. *Bacteroides* were closely associated with cancer-related metabolic pathways and may serve as potential diagnostic biomarkers.

Previous studies have demonstrated that PLWH and cancer patients exhibit varying degrees of gut microbiota dysbiosis (19). Our study reaffirmed that HIV infection significantly alters gut microbial diversity and further demonstrated that PLWH-C exhibited more severe dysbiosis, characterized by a marked reduction in microbial diversity and distinct community structures compared with PLWH-NC and Ctrl. Additionally, we characterized gut microbial features associated with HIV infection and various types of cancer. Random forest analysis identified 178 differential genera, including decreases in *Bacteroides* and *Prevotella*, an increase in *Escherichia-Shigella*, and 114 differential species. Notably, *Bacteroides vulgatus*—a common gut commensal—was the only species included in all of the diagnostic models. Among all detectable species applicable for diagnostic purposes, *Bacteroides* and *Prevotella* are the most prevalent. These genera are frequently reported in gut microbiota studies involving HIV and cancer. Existing evidence indicates that HIV infection is associated with reduced *Bacteroides* and enriched *Prevotella* abundance, both of which correlate with mucosal inflammation and immune dysregulation (20). In oncology contexts, *Bacteroides* may enhance immunotherapy responsiveness (21), whereas *Prevotella* could contribute to a pro-carcinogenic microenvironment (22). Nevertheless, strain-specific functional analyses remain to be further investigated. *B. vulgatus* is a common intestinal symbiotic bacterium and is also a member of the *Bacteroides* genus. Although it is often considered protective against CRC (23, 24), which is consistent with our findings, its role may vary depending on the strain and host context (25), suggesting that current understandings of host-microbiota interactions remain limited.

Further enrichment analysis using KEGG pathways revealed that *Bacteroides* and *Escherichia-Shigella* were closely associated with *Central carbon metabolism in cancer*, *Choline metabolism in cancer*, and *Proteoglycans in cancer*. Cancer-related metabolic pathways are closely interrelated, and the above three metabolic pathways are the primary metabolic pathways associated with cancer. Different types of cancer exhibit varying degrees of metabolic reprogramming. Glycolysis, as part of central carbon metabolism, is enhanced in most cancers, including CRC, lung cancer (26, 27). Abnormalities in choline metabolism are often associated with the development of non-small-cell lung cancer (28), whereas abnormalities in proteoglycan metabolism are more common in hepatocellular carcinoma (29). The 19 intersecting genera we screened for were significantly associated with all of these metabolic pathways, suggesting that disruption of these microbiota may lead to cancer-associated metabolic abnormalities in PLWH, which in turn affects cancer development. It is possible that a decrease in the number of the key bacteria we screened for (*Bacteroides* and *Prevotella*) leads to associated metabolic disorders that promote cancer development. It could also be an increase in key bacteria, such as *Escherichia-Shigella*, that promote specific metabolic processes and, thus, cancer progression. For example, studies showing a significant increase in the diversity and abundance of gut microbiota in patients with CRC, accompanied by changes in metabolites (30), as well as a significant decrease in *Lactobacillus* and *Bifidobacterium* and a higher prevalence of metabolic syndrome in patients with breast cancer suggest that a decrease in the gut microbiota may contribute to the development of breast cancer through metabolic disorders (31). One of the specific studies on CRC proposed that the specific regulatory mechanism of metabolism by microbiota may be the inhibition of glucose metabolism in colorectal cancer cells through the GPR109a-AKT or SIRT4/HIF-1α signaling pathways (32, 33). In addition, bacteria can also metabolize dietary choline, phosphatidylcholine, and carnitine to produce the trimethylamine-N-oxide precursor trimethylamine, which has been shown to have a pro-cancer effect in various cancers (34–36).

Finally, we constructed ROC models using the abundance table of the 19 intersecting bacteria to diagnose PLWH-KS, PLWH-C, PLWH-LC, and PLWH-CRC, respectively, with PLWH-NC and Ctrl. The results demonstrated that *Bacteroides* and its subspecies *B. vulgatus* exhibited moderate diagnostic performance. Numerous studies have demonstrated that the gut microbiota can serve as diagnostic biomarkers for various diseases (1, 4, 12, 37), offering valuable clinical insights for disease detection and diagnosis. Moreover, while the diagnosis of cancer is typically achieved through tissue biopsy, which is an invasive procedure, collecting gut microbiota from fecal samples is a noninvasive method. *Bacteroides*, in particular, hold promise as a novel approach for the detection of HIV-associated cancer in the future. This finding provides a theoretical foundation for developing new therapeutic strategies and treatment targets.

This study has several limitations. First, this study is limited by its cross-sectional design and small sample size, which restricts the generalization of the conclusions. In the future, we plan to conduct a longitudinal cohort study and increase the sample size. Second, PLWH who have cancer carry a substantial financial burden that can dampen their willingness to undergo viral load testing. In this study, roughly 35% of participants lacked viral load data, thereby limiting further analysis. We will strive to collaborate with local health institutions to foster greater participant motivation for completing viral load assessments and other related examinations. Additionally, we will enroll an HIV-negative cancer cohort and apply stricter inclusion criteria-such as matching CD4^+^ T cell counts, viral load levels, and antiretroviral therapy duration-to further refine the study design. Future investigations should also quantify HIV viral load, reservoir size (HIV DNA), transcriptional activity (HIV RNA), inflammatory markers, and gut metabolomic profiles in PLWH-C vs. PLWH-NC. Finally, as the present study is population-based and lacks experimental validation, we will integrate multi-omics analyses (metagenomics and metabolomics) and *in vitro* models (such as intestinal organoids) to delineate the interactions and mechanisms of the key bacterial species *B. vulgatus* in the context of cancer and HIV.

## Conclusions

In this study, we characterized the gut microbiota characteristics of PLWH, combined with KS, CRC, LC, and L, as well as PLWH-NC and Ctrl. For the first time, we revealed the microbial signatures associated with PLWH in cancer, highlighting a strong correlation between *Bacteroides* and cancer-related metabolic pathways. *Bacteroides* can serve as a diagnostic biomarker for HIV and various cancers, providing clinical insights into disease detection and diagnosis, as well as offering new scientific guidance for the development of probiotics.

## Data Availability

The raw fast data generated in this study were uploaded to the Sequence Read Archives (SRA) of the National Centre for Biotechnology Information (NCBI) under the BioProject PRJNA1108348. The PLWH-NC samples used in this study were taken from our previous BioProject PRJNA952811.
